# The impact of virtual learning on Multimedia University student performance: a cross-sectional study

**DOI:** 10.12688/f1000research.72881.1

**Published:** 2021-11-08

**Authors:** Tai Hen Toong, Lim Liyen, Liew Yee Ping

**Affiliations:** 1 Faculty of Business, Multimedia University Malaysia (MMU), Melaka, 75450, Malaysia; 2 Faculty of Information Science & Technology (FIST), Multimedia University (MMU), Melaka, 75450, Malaysia

**Keywords:** Virtual Learning, Student Performance, Covid-19 Pandemic

## Abstract

**Background: **The Covid-19 pandemic has imposed adaption to virtual learning for students and educators across all levels of education in the world. The effectiveness of virtual learning varies amongst age groups. It has been suggested that the adoption of virtual learning will continue to be implemented even after pandemic, particularly in higher education. Therefore, it is crucial to validate the effectiveness of a virtual learning approach among university students to ensure a smooth transition from a conventional education model to a hybrid education model. Thus, this study aims to evaluate the impact of virtual learning on students’ performance in a virtual classroom.

**Methods: **We analysed survey data collected from undergraduate students at Multimedia University, Malaysia. Convenience sampling and self-administered online surveys were used to understand the impact of virtual learning. Multiple regression analysis was performed using SPSS software

**Results:** A total of 210 first and second year degree and diploma students responded to the online surveys. Factors affecting virtual learning were segregated into three categories: virtual teaching techniques, technology issues, and environment distraction. Respondents stated that the critical factor that affect the effectiveness of virtual learning and impacts on students’ performance was the virtual teaching techniques employed by educators.

**Conclusions: **This study concluded that virtual teaching techniques have significant impact on students’ performance whereas technology issues and environment distraction do not significantly influence students’ performance during virtual learning. Although this study is limited to students from Multimedia University, it lays the groundwork for future research to involve students from other universities or other countries. A future study can address more factors that affect virtual learning and students’ performance, such as students’ attitude and motivation.

## F1000 Research Statement of Endorsement

Assoc. Prof. Dr. Ong Thian Song confirms that the author has an appropriate level of expertise to conduct this research, and confirms that the submission is of an acceptable scientific standard. Assoc. Prof. Dr. Ong Thian Song declares they have no competing interests. Affiliation: Multimedia University.

## Introduction

The Coronavirus disease-2019 (Covid-19) pandemic has affected many industries and sectors in terms of their operation and management worldwide. One of the greatly affected sectors is education. Since March 2020, 80% of the world’s leaners have been kept out of educational institutions due to the pandemic. ^1^ Educators and learners across all levels of education globally have therefore had to adapt to virtual learning during the pandemic. Transition from physical face-to-face classes to virtual remote learning has become new norm practice in many universities since the Covid-19 outbreak in Malaysia. It was reported that there are a total of 111 universities and colleges in Malaysia that were impacted significantly by the rapid spread of the Covid-19 virus since 2020. ^2^ Education institutions inclusive of schools and universities shifted towards the use of technologies and online learning during the pandemic period. ^3^ In Malaysia, 36.9% of students lacked access to online learning during the lockdown. ^4^ In most of the universities, academics were urged to implement virtual classroom with synchronous online teaching and learning in conjunction with closure of campus since the announcement of the Movement Control Order of 2020 to curb the spread of Covid-19. A synchronous learning environment is defined as “structured in the sense that students attend live lectures, there are real-time interactions between educators and learners, and there is a possibility of instant feedback”. ^5^ With synchronous online learning in a virtual classroom, student performance needs to be taken into consideration.

It has been suggested that adoption of virtual learning will continue to be implemented even after the pandemic particularly in higher education. There is a huge impact on universities towards virtual learning due to the abrupt and rapid conversion from the traditional classroom to a virtual learning environment in terms of the sustainability of the education model. ^6^ With regards to virtual learning, many universities also proposed hybrid learning when students are allowed to return to universities. Hybrid learning is referred to as “blended learning which lends itself to individualized learning, collaboration via online discussions, and several modes of interacting with course content for different learning styles”. ^7^ It is much more flexible and easier to incorporate and facilitate hybrid learning in a virtual learning environment. It is crucial to validate the effectiveness of a virtual learning approach among university students, to ensure a smooth transition from a conventional education model to online or hybrid education models.

The effectiveness of virtual learning with regards to students’ performance has not been explored among university students at our university, Multimedia University, in Malaysia. A prior study in public higher education institution in Portugal found that the implementation of virtual learning environments had imposed its influence on universities students’ performance. The study revealed that the higher frequency of access to virtual learning environment, students were more likely to pass the course. ^8^ Several studies have explored the differences between face-to-face classroom and online or hybrid learning, and the results revealed that students performed better in online or hybrid learning as compared to traditional education. ^9^^–^^11^ Thus, this paper aims to evaluate the impact of virtual learning towards students’ performance in a virtual classroom during the Covid-19 pandemic at our university.

### Virtual teaching techniques

Shifting from a traditional learning mode to virtual learning is not simple. There are a lot of factors that would bring a different impact or learning experience to the students. Based on the studies by Schmidt ^12^ and Kebritchi, ^13^ students’ learning experience is strongly impacted by the educators’ teaching style and the selection of online learning tools in their learning environment. Another study ^14^ shows that educators’ personality or attitude is one of the factors that would affect students’ learning motivation. The study revealed that the educator’s personalities such as being an extrovert, sensing, thinking, feeling and judging perspective do affect students’ learning motivation and it shows both positive and negative effects on the students’ learning experience. Another previous study ^15^ also studied the impact of personality traits towards the students’ online learning perceptions. The result shows that different personality traits do bring different impacts on students’ online learning perceptions. Educators’ self-knowledge in managing online learning tools plays an important role as well. Dubey and Singh ^16^ and Yu ^17^ have studied various factors on online teaching and found that young educators manage online education more actively and this may be due to self-technical knowledge or familiarity with online learning tools. Besides personality traits and self-knowledge, the educators’ voice and pitch are also considered as techniques in handling virtual classrooms, as they can be used a teaching tool to attract students’ attention during online learning. ^18^ A study by Bhowmik and Bhattacharya ^19^ has shown that educators’ personal factors such as immediate feedback, use of e-learning tools or trained well to handle online delivery highly influence students’ learning.

### Technology

During the pandemic, virtual learning became the main tool of education. Moving away from the traditional way of teaching and learning to virtual learning does provide flexibility, but it can also influence the students’ learning perception. Implementing virtual learning uses a wide range of ICT and technology-based teaching tools in teaching and learning. Previous research ^20^^–^^22^ has demonstrated the influence of learning with technology in the learning process. Another finding shows that there is correlation between the usage of Web 2.0 tools such as blogs, social networking sites and digital games with students in terms of learning engagement. ^23^ There are several studies being carried out on the ICT components that might affect the students’ learning experience such as uploading videos after the class, social media platforms such as YouTube or Facebook and usage of e-learning tools. ^24^^–^^26^ However, in some rural areas, issues, such as lack of devices and internet connection, can be found. According to Kulal and Nayak, ^27^ the increasing use of technology in online learning brings a positive impact to the students but it also emphasizes that lack of computers or mobile devices and network connection for rural students. It seems that even though educators have strong technical skills in utilizing the teaching-based technology, unstable internet connection remains the main concern. ^28^

### Environment distraction

Most studies are related to personality traits, technologies, usage of learning tools or lack of devices for learning, but there is another factor that brings impact to the students’ online learning process, which is environment distraction. Research on this factor is very limited compared to other factors. Several studies have been done in identifying what kind of distractions would influence students’ concentration in the class and they found that the students are easily distracted by the use of mobile devices such as cell phones. ^29^^,^^30^ Another study researched what makes students get distracted in the first place during the learning process, and found that it can be external factors (e.g. baby crying, people arguing in the same space) or internal factors (e.g. thinking of something, checking social networking sites). ^31^ Findings by Chhetri ^32^ and Coman et al ^33^ showed that there are distractions from surroundings or family members, which means it is hard to focus on learning. Another study by Realyvásquez-Vargas et al ^34^ also found that environmental conditions such as lighting, noise and temperature impact students’ learning performance.

### Students’ performance during virtual classroom

A lot of aspects may determine student performance in the virtual classroom. As shown by previous studies, ^35^^,^^36^ students show positive response to virtual learning in terms of communicating with educators and certain e-learning and game-based tools that involve interaction. As compared to physical classrooms, students have obtained better performance and scored higher grades through online learning. ^37^ In Ref. 38 study, the students prefer online learning when they are given the option and the result shows a higher rate of students scoring better through online learning. In the study by Paul and Jefferson, ^32^ it was found that lecture recordings from virtual classrooms are able to help students, especially if there are technical issues such as internet connection or surrounding distraction. Furthermore, there are several factors that have been identified such as feedback and online course design that might influence students’ performance during the online learning process. ^39^

## Methods

### Research framework

This paper’s framework shows the relationship among independent variables (virtual teaching techniques, technology, and environment distraction) and dependent variable (students’ performance during virtual classroom). Our hypotheses are provided below:

H1
**– Virtual teaching techniques** positively affect students’ performance during virtual classroom.

H2
**– Technology** positively affects students’ performance during virtual classroom.

H3
**– Environment distraction** positively affects students’ performance during virtual classroom.

**Figure 1.**
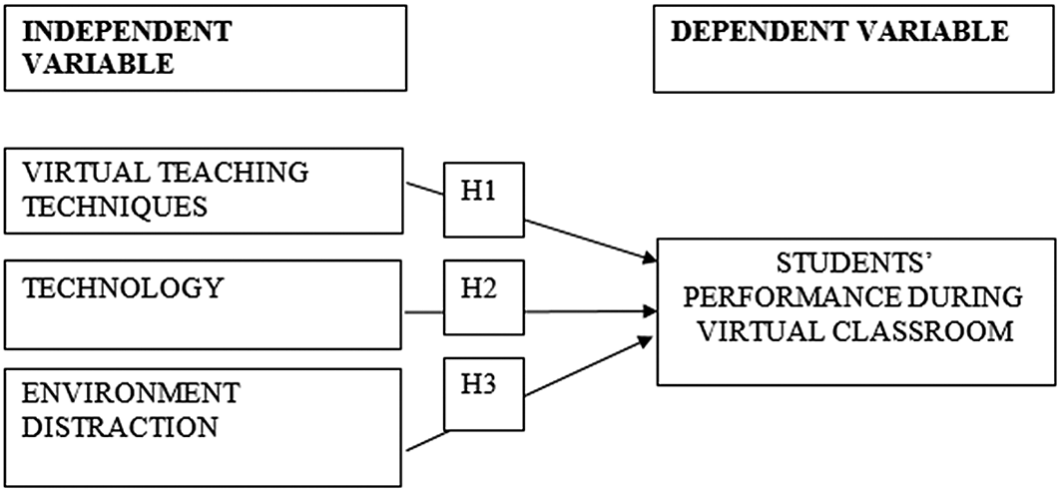
The research framework.

These three elements of independent variables and dependent variables might be able to construct a significant study for the higher education profession industry in Malaysia ( Figure 1).

Study setting and participants

The study population were IT students at Multimedia University. Convenience sampling was applied for respondent selection. Respondents were chosen from first year and second year students from the Degree and Diploma program, Faculty of Science and Technology at the university. Sample size comprises of 210 respondents.

Research Ethical Clearance was obtained from the Technology Transfer Office at our university (reference number: EA0242021). During data collection period, all participants were informed about the study and objectives. Participant consent was taken by incorporating the consent form within the questionnaire; respondents were asked to complete the consent part of the questionnaire before continuing. Questionnaires without the consent part filled in were not included in the analysis.

### Data collection

The online questionnaire was issued to the respondents in August 2020 for data collection. The questionnaire was comprised of three sections: Section A, four questions concerning respondent’s demographic profile (age, gender, first year students and programme enrolled) using closed-ended questions; Section B, three statements about the student’s perception on remote learning performance in virtual classrooms using Likert scales; Section C, five statements on the factors that affect their performance in the virtual classroom using Likert scales. A copy of the questionnaire can be found in the *Extended data.* ^46^

### Data analysis

IBM SPSS Statistic version 26 was used for data analysis. Statistical tests were as follows: Cronbach Alpha test, Pearson Correlation test and t-test. The standard level of significance was 5%, or stated as 0.05 in the p-value.

## Results

### Demographics

A total of 210 surveys were circulated in Multimedia University Malaysia among respondents aged between 20 to 30 years old. There was a 100% response rate. The biggest age group was <20 years old (n = 136, 64.8%), followed by 20-25 year olds (n = 73, 34.8%) and 25-30 year olds (n = 1, 0.5%). A total of 84.3% (n = 177) were male and 15.7% (n = 33) were female. The majority of the students who took part in the questionnaire are not from first year study in university. A total of 118 students (56%) were in their second or third year.

### Correlation between variables

Internal consistency reliability was assessed using Cronbach’s Alpha. The reliability is shown by a number that ranges from 0 to 1; values closer to 1 reveal higher reliability. All variables must achieve a critical value of greater than 0.75 to reflect a good internal reliability of the data. ^40^ Cronbach’s Alpha value for this questionnaire was 0.759, which is acceptable.

The three independent variables were tested for their relationship with students’ performance during virtual classroom. It was identified that there is a positive relationship between two variables (virtual teaching technique and technology), and there is a negative yet weak relationship between one variable (environment distraction). However, according to the result derived from Pearson correlation test ( Table 1) demonstrates that TE – Technology has a weak correlation with the impact of virtual learning towards students’ performance, which give a value of 0.234. Moreover, a weak negative/inverse relationship was been shown between EN – Environment Distraction with students’ performance during virtual classroom.

**Table 1.** Correlation between variables.

		I	VT	TE	EN
Students’ performance during virtual classroom	Pearson correlation	1			−0.073
	Significance level, p				0.290
Virtual teaching techniques	Pearson correlation	0.431	1		0.058
	Significance level, p	0.000			0.405
Technology	Pearson correlation	0.234	0.616	1	0.222
	Significance level, p	0.001	0.000		0.001
Environment distraction	Pearson correlation	−0.073	0.222	0.058	1
	Significance level, p	0.290	0.001	0.405	

To prepare variable has a strong relationship among dependent variables and independent variables, in this research the r value has to be provided with a value more than 0.5 according to Pearson correlation test. The highest correlated variable was virtual teaching techniques (Pearson’s Correlation = 0.431; p = 0.000), followed by technology (Pearson’s = 0.234; p = 0.001). Environment distraction showed a result of −0.073, a weak negative correlation with the impact of VT towards students’ performance during virtual classroom (p = 0.405). In sum, Pearson correlation analysis has demonstrated two variables are positively correlated with students’ performance during virtual classroom.

### Hypothesis testing

Table 2 demonstrates the multiple regression analysis used to test all the variables. It was found that virtual teaching techniques has a positive significance relationship with student performance during virtual classroom (p < 0.05).

**Table 2.** Regression analysis.

	B	T - stat	P – value
Constant	1.344	3.513	0.001
Virtual teaching techniques	0.557	5.644	0.000
Technology	−0.029	−0.275	0.784
Environment distraction	−0.082	−1.464	0.145
R ^2^		0.196	
Adjusted R ^2^		0.184	

Generally, a higher coefficient indicates a better fit of the model. The bigger the coefficient of Beta value, the stronger the determinant. Based on Table 2, the result demonstrates that the virtual teaching technique (B = 0.557) is the strongest predictor for the students’ performance during virtual classroom. As such, H1 is accepted. In contrast, technology and environment distraction showed insignificant and negative relationships with the students’ performance during virtual classroom, with B = −0.029 and −0.082, respectively. Moreover, the result demonstrated a P value of 0.784 and 0.145, respectively, which resulted in H2 and H3 both being rejected.

## Discussion

The research revealed that virtual teaching techniques have a notable impact on students’ performance in virtual classroom. In this regard, the response shows that lecturers’ teaching style, which incorporates collaborative learning and personalised learning during virtual learning, has an impact on their performance. In addition, students also believed that teaching techniques used by their lecturers to deliver the course lesson during virtual classroom positively affects their performance in the course. A previous study showed that if the teaching techniques are not matched with students’ learning techniques, it would impact on students’ academic performance. ^41^ Similarly, another study revealed that there is an impact of lecturers’ teaching styles towards the students’ academic performance. ^42^

However, the survey results showed insignificant and negative relationship between technology and its impact towards students’ performance in the virtual classroom. It showed that students do not recognize the significance of technological tools in relation to their performance. They do not perceive the importance of game-based learning or gamification learning with regards to their performance in the virtual classroom. According to previous studies, education environment with gamification resulted in poorer academic performance of university students. ^43^^,^^44^ The results of this study also indicated that environment distraction is negatively associated with the impact towards students’ performance in the virtual classroom. It showed that all students never consider the significance of background noise, like bird chirping, dog barking, building construction, road traffic and other distractions with regards to their performance in the virtual classroom. According to a previous study, loud noise during virtual learning can negatively affect students’ academic performance in a virtual classroom. ^45^

## Conclusions

Based on the analysis, this study concluded that IT students from our university believed that virtual teaching techniques significantly affect their performance in terms of online learning during a virtual classroom. Students appreciated the patience and sense of humour of lecturers during virtual classroom, which has significant impact on their performance. On the other hand, this study also concluded that technology and environment distraction do not show a significant impact on student performance of online learning during a virtual classroom. This could be due to IT students being already familiar with the usage of technological tools before the implementation of virtual learning and therefore the impact of technology applied during virtual classroom is not significant on their performance. Most of the IT students used a headset during the virtual classroom and that could be the reason that environment distraction does not significantly contribute to their performance of virtual learning. This study suggests that the lecturer applies an effective teaching style in virtual learning to improve students’ performance during the virtual classroom.

This study provides the groundwork for future research to involve students from other universities or other countries. Moreover, future study can address the impact of other factors which affect virtual learning and students’ performance such as students’ attitude and motivation. Future research can consider studying the significant difference between first year and non-first year students on factors affecting students’ performance in the virtual classroom.

## Data availability

### Underlying data

Figshare: An Empirical Study on the Impact of Virtual Learning on Multimedia University Student Performance.csv, https://doi.org/10.6084/m9.figshare.14872782.v4. ^46^

### Extended data

Figshare: An Empirical Study on the Impact of Virtual Learning on Multimedia University Student Performance.csv, https://doi.org/10.6084/m9.figshare.14872782.v4. ^46^

Data are available under the terms of the Creative Commons Attribution 4.0 International license (CC-BY 4.0).
