# Supplementary material for: The impact of virtual learning on Multimedia University student performance: a cross-sectional study
Source: F1000Res. 2021 Nov 8;10:1123. [Version 1] doi: 10.12688/f1000research.72881.1 (PMC8864185; doi:10.12688/f1000research.72881.1)
Supplement: Peer review history

# Peer review: Reviewer response for version 1

**DOI:** 10.5256/f1000research.76489.r122907

**Published:** 2022-02-22

**Authors:** Adrian A. Adascalitei

**Affiliations:** 1 Faculty of Electrical Engineering, “Gh. Asachi” Technical University, Iași, Romania

The Research Paper about the impact of virtual learning on Multimedia University student performance, is well realized. The paper evaluates the impact of virtual learning towards students’ performance in a virtual classroom.

 Factors affecting virtual learning were analysed and were segregated into three categories: virtual teaching techniques; technology issues; and environment distraction. Collaborative learning and personalised learning during virtual learning are essential. It is much more flexible and easier to incorporate and facilitate hybrid learning in a virtual learning environment.

 I congratulate the authors for the accomplishment of the study as well as the publication in the F1000Research journal.

Is the work clearly and accurately presented and does it cite the current literature?

Yes

If applicable, is the statistical analysis and its interpretation appropriate?

Yes

Are all the source data underlying the results available to ensure full reproducibility?

Yes

Is the study design appropriate and is the work technically sound?

Yes

Are the conclusions drawn adequately supported by the results?

Yes

Are sufficient details of methods and analysis provided to allow replication by others?

Yes

Reviewer Expertise:

Blended Teaching and Learning (Virtual Education)

I confirm that I have read this submission and believe that I have an appropriate level of expertise to confirm that it is of an acceptable scientific standard.

# Peer review: Reviewer response for version 1

**DOI:** 10.5256/f1000research.76489.r101024

**Published:** 2022-01-05

**Authors:** Francis Chuah

**Affiliations:** 1 School of Business Management, Universiti Utara Malaysia, Changlun, Malaysia

The study examines the impact of the virtual learning approach on Multimedia University students’ performance. A total of 210 first- and second-year degree and diploma students participated in the survey. I would like to congratulate the authors for the accomplishment of the study as well as the submission of the manuscript for publication in the F1000Research journal. While the manuscript is well written, I would like the authors to consider the following suggestions:

- I would recommend including a few more descriptive statistics information into the manuscript, i.e. assessment of normality and measures of distribution, and measures of central tendency so readers will have a clear view of the nature of the data for each variable.
- Both H2 and H3 show an insignificant relationship with the outcome variables, but in the discussion, the authors discuss the sign of the relationship as if the relationship is significant and negative. Why is that so? Statistically, it should be concluded that both technology and environmental distraction does not affect student performance in the classroom.

Is the work clearly and accurately presented and does it cite the current literature?

Yes

If applicable, is the statistical analysis and its interpretation appropriate?

Yes

Are all the source data underlying the results available to ensure full reproducibility?

Yes

Is the study design appropriate and is the work technically sound?

Yes

Are the conclusions drawn adequately supported by the results?

Yes

Are sufficient details of methods and analysis provided to allow replication by others?

Yes

Reviewer Expertise:

Virtual learning, statistical analysis

I confirm that I have read this submission and believe that I have an appropriate level of expertise to confirm that it is of an acceptable scientific standard.
